# Methylmalonic acid, vitamin B12, and mortality risk in patients with preexisting coronary heart disease: a prospective cohort study

**DOI:** 10.1186/s12937-023-00900-6

**Published:** 2023-11-29

**Authors:** Junchen Guo, XiaoXuan Liu, Zeng Wang, Rongzhe Lu, Yige Liu, Yiying Zhang, Wei Tian, Shaohong Fang, Shanjie Wang, Bo Yu

**Affiliations:** 1 Department of Cardiology, Second Affiliated Hospital of Harbin Medical University, Harbin, 150000 China; 2 The Key Laboratory of Myocardial Ischemia, Chinese Ministry of Education, Harbin, 150000 China; 3 Department of Epidemiology and Biostatistics, School of Public Health, Harbin Medical University, Harbin, 150000 China; 4 Department of Epidemiology and Biostatistics, School of Public Health, Jiamusi University, Jiamusi, 154000 China

**Keywords:** Methylmalonic acid, Vitamin B12, Biomarker, Mortality, Coronary Heart Disease

## Abstract

**Background:**

The inconsistent relationship between Vitamin B12 (B12), methylmalonic acid (MMA, marker of B12 deficiency) and mortality was poorly understood, especially in patients with coronary heart disease (CHD). This study aims to investigate the association of serum MMA, and B12-related biomarkers (serum level, dietary intake, supplement use, and sensibility to B12) with all-cause and cardiovascular mortality in adults with CHD.

**Methods:**

The data of this study were from a subcohort within the US National Health and Nutrition Examination Survey (NHANES). We included adults with preexisting CHD with serum MMA and B12, and dietary B12 intake measurements at recruitment. All participants were followed up until 31 December 2019. Weighted Cox proportional hazard regression was used to estimate hazard ratios (HR) and 95% CI of mortality risk.

**Results:**

Overall, 1755 individuals (weighted mean [SE] age, 65.2 [0.5] years; 1047 men [weighted 58.5%]) with CHD were included, with geometric mean levels of serum MMA 182.4 nmol/L, serum B12 494.5 pg/ml, and dietary B12 intake 4.42 mg/day, and percentage of B12 supplements use 39.1%. During a median follow-up of 7.92 years, 980 patients died. Serum B12 concentration, dietary B12 intake and supplements use were not significantly associated with mortality risk (each p ≥ 0.388). In contrast, individuals in the top tertile of MMA had multivariable-adjusted HRs (95% CIs) of 1.70 (1.31–2.20) for all-cause mortality, and 2.00 (1.39–2.89) for cardiovascular mortality (both p trend < 0.001) compared to those in the bottom tertile of MMA. MMA-related mortality risk was particularly higher among participants with sufficient serum B12 (p < 0.001). CHD patients with increased levels of both MMA and B12 had a doubled mortality risk compared to those with lower MMA and B12 (p < 0.001).

**Conclusion:**

MMA accumulation but not serum or dietary vitamin B12 was associated with increased cardiovascular mortality risk among patients with CHD. This paradox may be related to decreased response to vitamin B12.

**Supplementary Information:**

The online version contains supplementary material available at 10.1186/s12937-023-00900-6.

## Introduction

Coronary heart disease (CHD) is responsible for 15.6% of all deaths worldwide, making it one of the leading causes of mortality [1]. Traditional risk factors, including hypertension, smoking, diabetes, physical inactivity, obesity, high blood cholesterol, depression, and excessive alcohol consumption, can only partially explain the disease burden of CHD [2]. Vitamin B12 (B12) is an essential nutrient for nucleotide and amino acid biosynthesis [3–5]. Although B12 deficiency has been associated with cardiovascular disease (CVD) and diabetes, the benefits of increased B12 levels were controversial. For example, some evidence demonstrated that higher blood concentrations of vitamin B12 were associated with an increased risk of adverse events [3, 6–8]. Our recent study observed that serum and dietary B12 were not significantly associated with reduced mortality risk in adults with diabetes [9, 10]. Consistently, previous clinical trials found no significant cardiovascular benefits of B12 supplements use in subjects with vascular disease [11, 12]. Overall, mortality benefits of B12 management in CHD was unclear.

In contrast, we previously found that serum methylmalonic acid (MMA), an established biomarker of B12 deficiency, was robustly associated with an increased risk of cardiovascular mortality in the general population and diabetic patients [9, 10]. The Western Norway Coronary Angiography Cohort (WECAC) study recently reported the association between plasma MMA, and risk of acute myocardial infarction (AMI) and mortality in patients with suspected stable angina pectoris [13]. However, B12 was not considered in this analysis. Notably, a paradox condition of increase in both MMA and B12 might be explained by the decreased sensitivity to B12 treatment [10]. For instance, one of the most common causes of congenital methylmalonic acidemia is a point mutation leading to inactivation of the mitochondrial enzyme methylmalonyl-CoA mutase (MMUT), which results in no response to treatment with B12 [14, 15].

To date, no study has assessed the association between B12 sensitivity and mortality risk in CHD. Understanding this knowledge may provide some new insights into B12 management in patients with CHD. In addition to increasing B12 supplementation, whether improving sensitivity to B12 is a more effective strategy for promoting cardiovascular health. To address these gaps, we comprehensively investigated the associations of serum MMA and B12-related biomarkers (serum B12 level, dietary B12 intakes from foods, B12 supplements use, and sensitivity to B12) with risks of cardiovascular and all-cause mortality in a nationally representative sample of US adults with CHD.

## Method

### Study population

National Health and Nutrition Examination Survey (NHANES) is an ongoing program of studies that combines interviews and physical examinations in the form of a series of surveys, aiming to assess the health and nutritional status of adults and children in the United States. We analyzed data of the NHANES cycles from five study cycles (1999 to 2000, 2001 to 2002, 2003 to 2004, 2011 to 2012, and 2013 to 2014) because both serum B12 and MMA were only measured in those study cycles. Overall, among 26,661 adults aged 20 years, there was a total of 2072 subjects with past histories of CHD. After excluding without serum MMA and B12 measurement (n = 315) and lost to follow-up (n = 2), 1755 eligible patients were included in our analysis.

### Study exposure: MMA and vitamin B12

Blood samples were collected via venipuncture, and MMA was determined in plasma and/or serum. MMA level was measured using Gas chromatography-mass spectrometry (GC/MS) for the 1999 through 2004 surveys, and Liquid chromatography-mass spectrometry (LC-MS/MS) for the 2011–2014 surveys. An evaluation of MMA measurement by two methods (n = 326) showed excellent correlation (r = 0.99) and consistency (Deming regression, Bland-Altman analysis) for GC/MS and LC-MS/MS detection, supporting that MMA data measured by both protocols can be combined for analysis.

Serum B12 levels were determined using the Quantaphase II Folate/B12 Radioassay Kit (Bio-Rad Laboratories) between 1999 and 2004, and an automated electrochemiluminescence immunoassay (Elecsys E170; Roche) between 2011 and 2014 in the central laboratory of NHANES. Both assays demonstrated comparable coefficients of variation (< 5%) and limits of detection (20–30 pg/mL). A comparative analysis was conducted in-house on 284 specimens to evaluate any differences between the two assays. To account for errors considered by Deming regression in both methods, B12 values obtained using the Roche assay were converted to Bio-Rad B12 levels based on NHANES recommendations.

Dietary intake was assessed by 24-h food recalls conducted by trained interviewers. From 1999 to 2002, one diet recall was conducted in-person in the Mobile Examination Center; since 2003, the second recall was added via telephone 3 to 10 days later after the first recall and the average of the two dietary recall was adopted to reduce estimation errors [10]. Standard protocols and tools were employed to aid in assessing the volume and dimensions of the food consumed. The Food and Nutrient Database for Dietary Studies was utilized to estimate the nutritional components of the foods [16].

Information regarding the consumption of dietary supplements containing B12 was obtained through standardized questionnaires [16]. All participants were queried about their use of dietary supplements within the previous 30 days. To minimize misclassification errors, the ingredient information was verified by referencing the bottles and labels.

Functional B12 deficiency, characterized by impaired sensitivity to B12 therapy, has been previously defined as MMA > 250 nmol/L and serum B12 > 400 pg/mL [17, 18]. In this study, patients with CHD were categorized into four groups based on the combination of binary serum B12 and MMA, enabling the assessment of B12 sensitivity. The groups included MMA_low_B12_low_ (MMA ≤ 250 nmol/L, B12 ≤ 400 pg/mL), MMA_low_B12_high_ (MMA ≤ 250 nmol/L, B12 > 400 pg/mL), MMA_high_B12_low_ (MMA > 250 nmol/L, B12 ≤ 400 pg/mL), and MMA_high_B12_high_ (MMA > 250 nmol/L, B12 > 400 pg/mL).

### Covariates

The following variables were collected by standardized questionnaires: age, sex, race/ethnicity, smoking status, alcohol consumption, and physical activity. Race/ethnicity was recorded as non-Hispanic whites, non-Hispanic blacks, Hispanic Mexicans and others. Smoking status was categorized as never, former and current smokers. Former smokers were defined as participants who had ever smoked at least 100 cigarettes but had now quit. Alcohol consumption (g/d) was estimated according to questionnaire. One drink, approximately 10 g of alcohol, was defined as 12-oz beer, 4-oz wine, or 1 ounce of liquor [19]. Leisure physical activity status (inactive, moderate, and vigorous) was determined by the self-reported [19]. Moderate physical activity was defined as leisure activities with a light sweating or a slight to moderate increase in breathing or heart rate in the past month. Vigorous activity was defined as exercise with heavy sweating, or significant increase in breathing or heart rate for at least 10 min [19]. Medications use, including cardiovascular prescriptions ACEI/ARB, β-blocker, diuretics, anti-lipid agents, and anti-platelet agents, and Metformin, during the 30 days prior to the interview was ascertained from self-report [20]. The specific names of prescription drugs were ascertained according to the medication container label, which was linked to standardized generic prescription medication catalogues, as our previously described [21]. Body mass index (BMI) was calculated as weight (in kilograms) divided by height (in meters) squared. The estimated glomerular filtration rate (eGFR) was calculated using the Chronic Kidney Disease Epidemiology Collaboration equation. The total cholesterol (TC), high-density lipoprotein cholesterol (HDL-C), and C-reactive protein were measured as our previous described [9]. Hypertension was defined as antihypertensive prescription medication, systolic BP ≥ 140 mmHg, or diastolic BP ≥ 90 mmHg. Diabetes was defined as treatment with anti-hyperglycemic or HbA1c ≥ 6.5%. Chronic obstructive pulmonary disease (COPD) including chronic bronchitis and/or emphysema were defined according to the self-reported diagnosis [9]. Malignancy or cancer was recorded by a self-report diagnosed by doctor.

### Study outcomes

The study endpoints were all-cause mortality, cardiovascular mortality, heart-specific mortality, stroke-related mortality and cancer-related mortality. Mortality status and cause of death were determined by NHANES-linked National Death Index public access files through December 31, 2019. The leading cause of death was identified based on the International Classification of Diseases, 10th Revision (ICD-10), including death due to cardiovascular disease (heart disease: I00-I09, I11, I13, I20-I51, and cerebrovascular disease: I60-I69), malignant neoplasms (C00-C97). Because of International Classification of Diseases, 9th Revision (ICD-9) used prior to 2015, we translated from ICD-9 code to ICD-10 code for further analysis.

### Statistical analysis

All analyses were conducted in compliance with the analytical guidelines of the NHANES data set [10]. All estimates are weighted with a raw masked variance of primary sampling unit, pseudo-strata, and appropriate sampling weights to account for complex sampling designs, unless otherwise stated. Continuous variables and categorical variables are expressed as weighted mean (standard error, SE), and weighted percentages, respectively. Participants with CHD were divided into three tertiles groups: T1 (MMA < 120 nmol/L), T2 (MMA 120–216 nmol/L), and T3 (MMA > 216 nmol/L). The trend test was used to analyze the baseline characteristics across MMA groups. Weighted linear regression was used for measurement data, and logistic regression was used for categorical variables.

Estimates of all-cause mortality, cause-specific mortality, and 95% CI are based on the Poisson distribution, where mortality is expressed as the number of deaths per 1000 person-years of follow-up. If an intersection point is identified, we further conducted the Landmark analysis to assess to the mortality risk occurring before and after the intersection point. Unadjusted and multivariate adjusted weighted Cox proportional risk regressions were used to assess the association between MMA, B12-related biomarkers (serum level, dietary intakes, supplements use, and sensitivity to B12), and the risk of mortality. Three adjustment models are used. Model 1 was adjusted for age (years, continuous variables), sex (male, female), and race/ethnicity (non-Hispanic white, black, Hispanic Mexican, or other). Model 2 was additionally adjusted for smoking status (never, ever, or current), physical activity (inactive, moderate, or vigorous), body mass index (< 18.5, 18.5–25, 25–30, or ≥ 30 kg/m^2^), hypertension (no/yes), diabetes (no/yes), chronic obstructive pulmonary disease (no/yes), cancer (no/yes), total cholesterol (mmol/L, continuous), high-density lipoprotein cholesterol (mmol/L, continuous), C-reactive protein (mg/dL, continuous), Vitamin B12 (B12, continuous), and estimated glomerular filtration rate (mL/min/1.73 m², continuous) were added. Model 3 was additionally adjusted for cardiovascular medications, including metformin (no/yes), ACEI/ARB drugs (no/yes), β-blocker use (no/yes), diuretic drugs (no/yes), anti-lipid use (no/yes) and anti-platelet use (no/yes), was also adjusted. The hazard ratio was calculated using the first group of classification variables (T1) as reference. Models for B12 supplements use were adjusted for dietary B12 intake from foods (continuous) and vice versa.

The association between baseline MMA and all-cause mortality was further stratified into specific subgroups based on age (< 65 years and ≥ 65 years), sex (male/female), current smoking (no/yes), body mass index (< 30 and ≥ 30), diabetes (no/yes) and eGFR (< 60 mL/min/1.73m^2^ and ≥ 60 ml/min/1.73m^2^) were grouped. The Survey-Weighted Wald test was used to assess the potential interaction between MMA and stratification factors on the risk of death. ROC curve analysis confirmed the prognostic performance of MMA and other cardiovascular biomarkers in patients with CHD. A two-tailed P value of < 0.05 was considered statistically significant.

## Results

### Baseline characteristics

Overall, among 26,661 adults aged 20 years, 2072 subjects with past histories of CHD. After excluding without serum MMA and B12 measurement (n = 315) and lost to follow-up (n = 2), 1755 adults were included in the analysis (Supplementary Fig. 1). Geometric mean level of serum MMA was 182.4 nmol/L, serum B12 was 494.5 pg/ml, and dietary B12 intake from foods was 4.42 mg/day, and 39.1% participants used supplements containing B12. Patients were divided into three tertiles groups based on their MMA levels: T1 (MMA < 120 nmol/L), T2 (MMA 120–216 nmol/L), and T3 (MMA > 216 nmol/L) (Table 1). Baseline characteristics showed that participants with higher MMA levels were older. Patients with higher MMA levels were more often taking less exercise and suffering from comorbidities, such as diabetes and cancer. Although Serum B12 showed a downward trend with the increase of MMA level, B12 in most patients is still higher than the cutoff value of B12 deficiency (< 203 pg/mL). Among patients with MMA > 250 nmol/L, about 47% had serum B12 more than 400 pg/mL.

**Table 1:** Baseline characteristics of participants

	Circulating MMA, nmol/L			
Variables	T1 (n = 592)	T2 (n = 579)	T3 (n = 584)	p trend
Age, years^*^	59.7 ± 0.69	66.7 ± 0.73	69.4 ± 0.71	< 0.001
Sex, %				0.08
Female	242 (39.2)	220 (39.5)	248 (46.6)	
Male	351 (60.8)	360 (60.5)	336 (53.4)	
Race/ethnicity, %				
Non-Hispanic White	281 (73.5)	381 (82.9)	402 (83.6)	< 0.001
Non-Hispanic Black	135 (12.4)	81 (6.3)	72 (6.1)	< 0.001
Hispanic-Mexican	109 (5.4)	60 (3.3)	59 (2.4)	0.003
Other Ethnicity	68 (8.7)	58 (7.5)	51 (7.9)	0.063
BMI, kg/m^2^	30.1 ± 0.39	29.6 ± 0.31	29.3 ± 0.39	0.193
Smoking status				0.028
Never	223 (35.2)	226 (38.9)	237 (40.0)	
Former	241 (41.1)	250 (40.5)	264 (44.5)	
Current	129 (23.7)	104 (20.6)	83 (15.5)	
Alcohol consumption, g/day	3.9 ± 0.41	5.4 ± 1.79	2.1 ± 0.27	< 0.001
Physical activity, %				< 0.001
Inactive activity	323 (49.1)	340 (55.0)	403 (65.7)	
Moderate activity	163 (30.4)	183 (33.1)	154 (27.8)	
Vigorous activity	107 (20.5)	57 (11.9)	27 (6.5)	
Hypertension	431 (32.5)	431 (27.5)	481 (20.0)	0.002
Total cholesterol, mmol/L	4.9 ± 0.07	4.9 ± 0.07	4.7 ± 0.06	0.03
HDL-C, mmol/L	1.24 ± 0.02	1.3 ± 0.03	1.2 ± 0.02	0.497
CRP, mg/dl	0.6 ± 0.05	0.5 ± 0.04	0.6 ± 0.05	0.875
eGFR, ml/min per 1.73 m²	89.0 ± 0.90	73.5 ± 0.95	61.0 ± 1.31	< 0.001
Diabetes, %	182 (24.6)	155 (22.6)	222 (34.9)	0.003
COPD, %	85 (6.4)	88 (7.7)	108 (9.0)	0.332
Cancer, %	81 (17.3)	166 (17.6)	132 (20.1)	< 0.001
B12, pg/mL	638.7 ± 26.88	579.4 ± 21.12	518.9 ± 19.28	< 0.001
ACEI/ARB, %	261 (45.0)	272 (43.3)	278 (46.0)	0.821
β-blocker, %	222 (36.8)	285 (49.0)	290 (50.8)	< 0.001
Diuretics, %	133 (19.8)	188 (28.3)	276 (47.2)	< 0.001
Anti-lipid agents, %	286 (52.7)	346 (62.8)	324 (59.4)	0.067
Anti-platelet agents, %	83 (13.4)	95 (18.0)	103 (17.6)	0.094
Metformin, %	80 (12.3)	68 (11.0)	61 (9.3)	0.216

Continuous variables are presented as mean ± standard error. Categorical variables are presented as number (%). The number for categorical variables was unweighted. All estimates were adjusted for survey weights of NHANES. P for trend was estimated with linear regression for continuous variables and with logistic regression for categorical variables. BMI: body mass index; COPD: chronic obstructive pulmonary disease; eGFR: estimated glomerular filtration rate; CRP: C-reactive protein; HDL-C: high-density lipoprotein cholesterol; B12: Vitamin B12

As shown in Supplementary Table 1, MMA was positively correlated with Creatinine, Blood urea nitrogen, and C-Peptide (r = 0.388, 0.293, and 0.229, respectively), while inversely correlated with High-density lipoprotein-cholesterol (HDL-C), eGFR, and B12 (r = -0.102, -0.405, and − 0.291, respectively). However, the correlation with BMI, Waist circumference, Glucose, HbA1c, Insulin, HOMA-IR index, triglycerides (TG), total cholesterol (TC), Low-density lipoprotein-cholesterol (LDL-C), Systolic BP, C-reactive protein (CRP) and Serum folate was insignificant or weak.

### The association between mitochondria-derived metabolite MMA and mortality risk

**Fig. 1:**
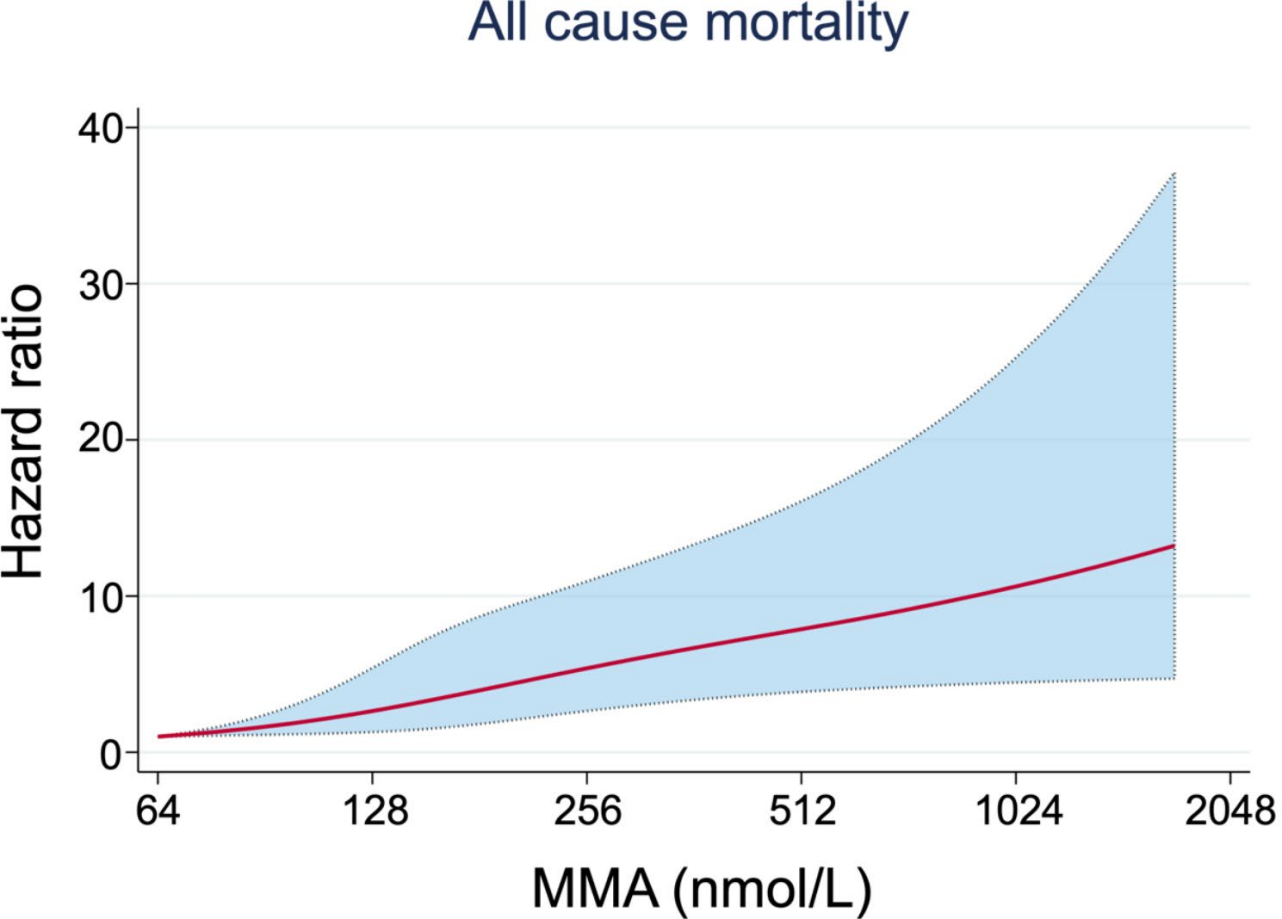
The restricted cubic spline showing the association of MMA with all-cause mortality risk The restricted cubic spline curve shows the association of MMA and all-cause mortality. Y-axis shows Hazard Ratio estimated by a univariate Cox proportional regression model. The X-axis shows the MMA content (nmol/L). The solid and dashed lines represent point estimates 95% CIs, respectively

**Fig. 2:**
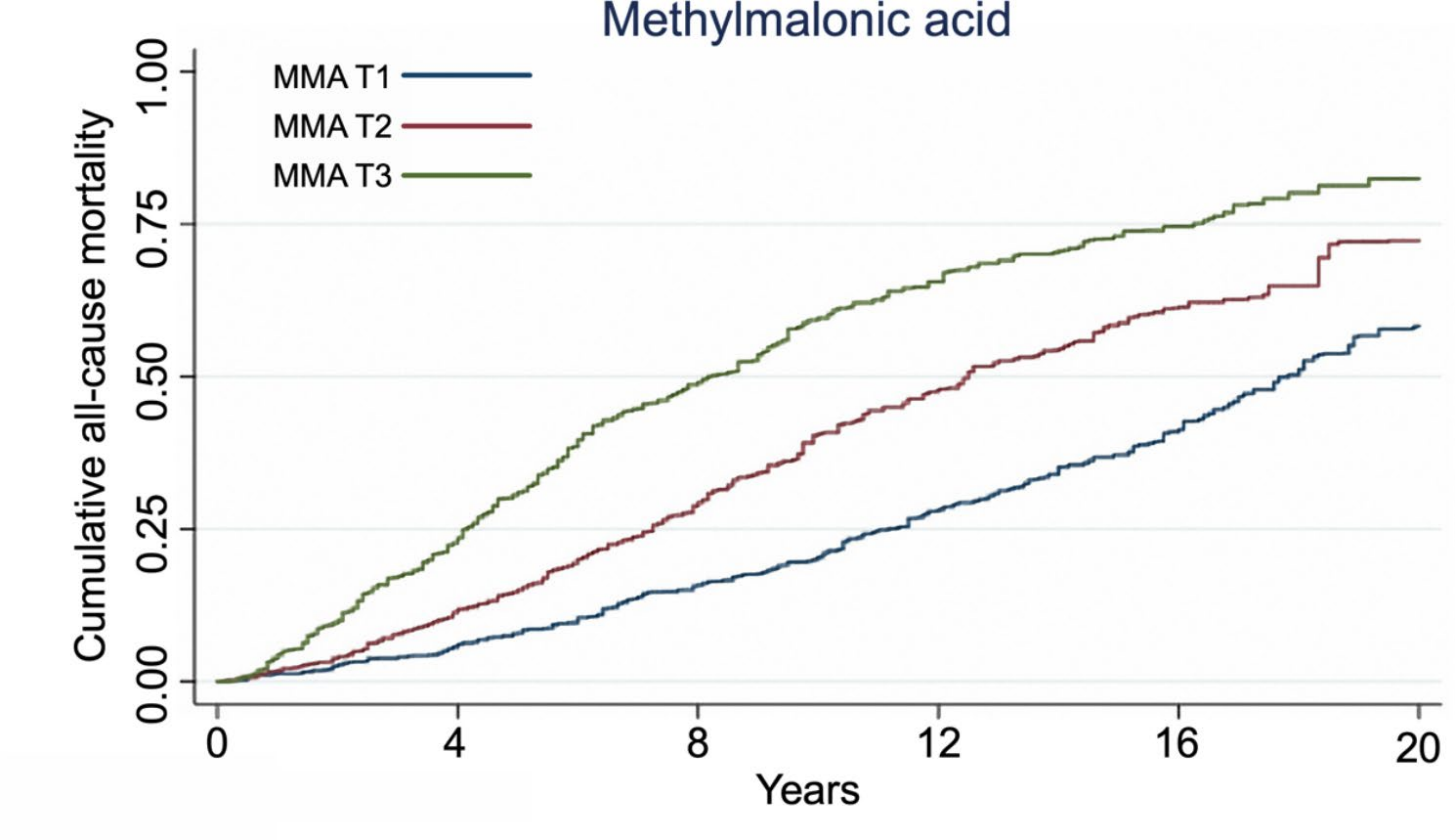
Accumulative all-cause mortality across methylmalonic acid strata in patients with coronary heart disease Accumulative all-cause mortality in NHANES 1994–2014 across the three tertiles groups during follow-up. P value was estimated by log-rank test. Y-axis shows the absolute risk of cumulative mortality rate (%). The X-axis shows the follow-up period (years)

During the median follow-up of 7.92 years, 980 (49.6%) patients died. Restricted cubic spline showed the linear relationship between serum MMA and risk of all-cause mortality (Fig. 1). The cumulative mortality rates per 1000 person-years for all-cause was 37.60, 61.46 and 102.62 for patients with MMA levels in T1, T2 and T3, respectively (Fig. 2). According to Kaplan-Meier plots, patients with elevated levels of MMA had significantly increased cumulative incidence of cardiovascular and heart-specific death (Supplementary Fig. 2). Elevated value of serum MMA was robustly associated with all-cause mortality in Cox regression models (Table 2). After adjustment for traditional cardiovascular risk factors and B12, we also observed a nearly two-fold increased risk of death for patients with MMA values in T3 (HR 1.79; 95% CI 1.39 to 2.30, p < 0.001) compared with the reference T1 group. When we considered cardiovascular drug use (model 3), the relationship remained significant (HR 1.70, 95% CI 1.31 to 2.20, p < 0.001). In addition, we observed a similar trend in cardiovascular mortality (HR 2.00, 95% CI 1.39 to 2.89, T3 versus T1, p < 0.001) and heart-specific disease (HR 1.94; 95% CI 1.31 to 2.87, T3 versus T1, p = 0.001) (Table 2). However, the relationship between MMA and mortality due to stroke or cancer was insignificant (Supplementary Table 2).

**Table 2:** The association of methylmalonic acid with all-cause mortality and cardiovascular mortality

			MMA, nmol/L			
Cause of death	log MMA* (n = 1755)	p value	T1 (n = 592)	T2 (n = 579)	T3 (n = 584)	p trend
**All-cause**						
Deaths/ person-yrs&	980/15,877	-	255/6782	311/5060	414/4034	-
Crude	2.10 (1.80–2.46)#	< 0.001	1 (ref.)	1.66 (1.34–2.06)	2.87 (2.36–3.46)	< 0.001
Model 1	1.64 (1.41–1.93)	< 0.001	1 (ref.)	1.14 (0.94–1.40)	1.87 (1.50–2.32)	< 0.001
Model 2	1.83 (1.56–2.16)	< 0.001	1 (ref.)	1.12 (0.88–1.41)	1.79 (1.39–2.30)	< 0.001
Model 3	1.78 (1.51–2.12)	< 0.001	1 (ref.)	1.11 (0.88–1.41)	1.70 (1.31–2.20)	< 0.001
**CVD**						
Deaths/ person-yrs	425/15,877	-	98/6782	144/5060	183/4034	-
Crude	2.19 (1.83–2.62)	< 0.001	1 (ref.)	2.05 (1.39–3.02)	3.30 (2.48–4.40)	< 0.001
Model 1	1.71 (1.41–2.08)	< 0.001	1 (ref.)	1.39 (1.00-1.93)	2.10 (1.57–2.79)	< 0.001
Model 2	2.20 (1.71–2.84)	< 0.001	1 (ref.)	1.40 (0.94–2.06)	2.15 (1.52–3.05)	< 0.001
Model 3	2.10 (1.61–2.74)	< 0.001	1 (ref.)	1.38 (0.93–2.06)	2.00 (1.39–2.89)	< 0.001
**Heart disease**						
Deaths/ person-yrs	369/15,877	-	88/6782	120/5060	161/4034	-
Crude	2.24 (1.86–2.72)	< 0.001	1 (ref.)	1.97 (1.32–2.95)	3.30 (2.46–4.43)	< 0.001
Model 1	1.79 (1.46–2.20)	< 0.001	1 (ref.)	1.36 (0.96–1.93)	2.14 (1.59–2.89)	< 0.001
Model 2	2.30 (1.76–3.02)	< 0.001	1 (ref.)	1.36 (0.90–2.05)	2.11 (1.46–3.05)	< 0.001
Model 3	2.18 (1.63–2.91)	< 0.001	1 (ref.)	1.34 (0.88–2.04)	1.94 (1.31–2.87)	0.001

Ref, treating the bottom group (MMA < 120nmol/L) as the reference; CVD, cardiovascular disease;

*Hazard ratio per 1 unit increases of natural log-transformed MMA; & unweighted; # Values are weighted hazard ratio (95% confidence interval)

Model 1: adjusted for age (years, continuous), sex (female or male), and race/ethnicity (non-Hispanic white, black, Hispanic-Mexican, or other)

Model 2: additionally adjusted for smoking status (never, ever or current), physical activity (inactive, moderate, or vigorous), body mass index (< 18.5, 18.5–25, 25–30, or ≥ 30 kg/m^2^), hypertension (no/yes), diabetes (no/yes), chronic obstructive pulmonary disease (no/yes), cancer (no/yes), total cholesterol (mmol/L, continuous), High-density lipoprotein cholesterol (mmol/L, continuous), C-reactive protein (mg/dL, continuous), Vitamin B12 (B12, continuous) and estimated glomerular filtration rate (ml/min/1.73 m², continuous)

Model 3: additionally adjusted for metformin use (no/yes), ACEI/ARB use (no/yes), β-blocker use (no/yes), diuretics use (no/yes), anti-lipid use (no/yes) and anti-platelet use (no/yes)

Stratified analysis showed that the association between MMA levels and mortality risk was largely consistent across subgroups (Supplementary Table 4). Notably, we did observe a more pronounced effect among patients with diabetes compared to those without diabetes (p for interaction = 0.019). Receiver Operating Characteristic (ROC) curve analysis confirmed the predictive performance of MMA and CRP for CHD prognosis (Supplementary Fig. 3). MMA, with a higher area under the ROC curve (0.723), demonstrated superior performance compared to CRP (0.557).

### The insignificant association between serum B12, B12 intake from food, B12 supplement use and mortality risk

**Fig. 3:**
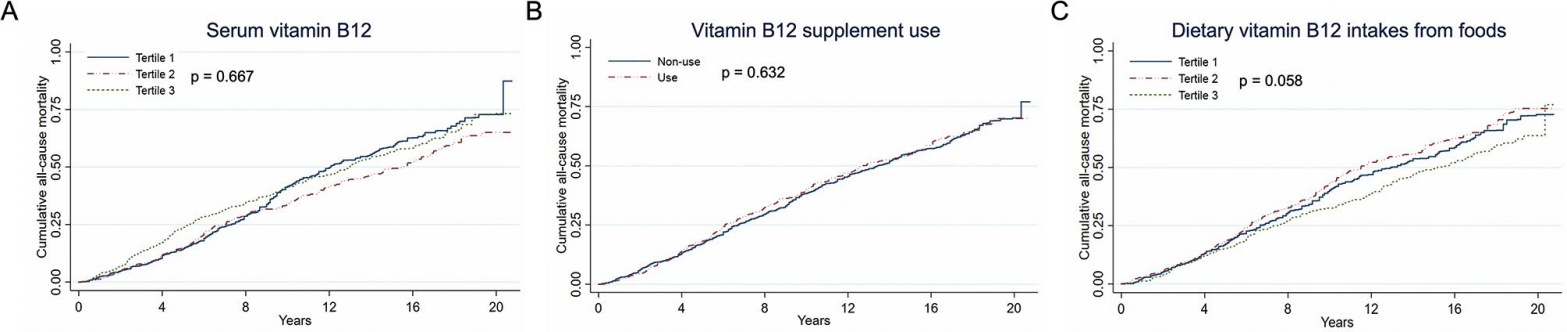
Accumulative all-cause mortality by serum B12, B12 supplement use and B12 dietary intake from foods **A:** Serum vitamin B12. **B:** Vitamin B12 supplements. **C:** Vitamin B12 intake from foods. Y-axis shows the absolute risk of cumulative mortality rate (%). P value was estimated by log-rank test. The X-axis shows the follow-up period (years). Proportional hazards assumptions were met by B and C but not by A

Serum B12 levels were examined for their association with all-cause mortality in patients with CHD by a series of Cox proportional hazards regression analyses and weighted Kaplan-Meier plots (Fig. 3 and Supplementary Table 3). However, no significant associations were found. When comparing the highest tertile of serum B12 to the lowest tertile (model 3), the multivariable-adjusted hazard ratios HRs and 95% confidence intervals CIs were 0.98 (0.80–1.20) for all-cause mortality (P = 0.835). According to the intersection point, we further conducted the Landmark analysis to assess the mortality risk occurring before and after 9 years of follow-up across tertiles of serum vitamin B12. There is a borderline significance between groups during the 9-year follow-up period (p = 0.048) (Supplementary Fig. 4). Additionally, the potential impact of different sources of B12 intake was considered. Patients with higher B12 intake from food sources showed no significant reduced risk of all-cause mortality in adjusted model 3 (T3 vs. T1: HR 0.91 [95% CI 0.73–1.14], P = 0.388). Consistently, the use of B12 supplements did not significantly reduce the risk of all-cause mortality (HR 0.97 [95% CI 0.81–1.15], P = 0.699) when compared to nonusers after further considering cardiovascular drug use (model 3).

### Declined sensitivity to B12 was related to increased risk of mortality

Firstly, we initially investigated whether the association between increased mortality and MMA levels varied among patients with high or low serum B12 levels (Table 3). A significant interaction was observed between serum B12 and MMA in relation to all-cause mortality (P < 0.025). The adjusted HRs and 95% CIs per doubling of MMA for all-cause mortality were 1.54 (1.21–1.95) among patients with B12 < 400 pg/mL and 2.14 (1.64–2.79) among those with B12 ≥ 400 pg/mL. Cox proportional hazards regression analyses demonstrated that patients with both MMA > 250 nmol/L and B12 > 400 pg/mL exhibited the highest risk of all-cause mortality, even after adjusting for the mentioned covariates (Table 3). When compared to patients with low MMA and low B12 (MMA_low_B12_low_), the multivariate-adjusted HRs (95% CIs) for total mortality in the MMA_low_B12_high,_ MMA_high_B12_low_, and MMA_high_B12_high_ groups were 0.90 (0.73–1.12), 1.27 (0.95–1.69), and 1.98 (1.46–2.69), respectively.

**Table 3:** The associations between declined response to B12 and mortality risk in patients with CHD

		MMA-associated mortality in Cbl subgroups		Combination of binary MMA and Cbl			
		Cbl < 400 pg/ml	Cbl ≥ 400 pg/ml	MMA_low_ Cbl_low_	MMA_low_ Cbl_high_	MMA_high_ Cbl_low_	MMA_high_ Cbl_high_
**Unadjusted**	HR (95%CI)	1.81 (1.47–2.21)	2.95 (2.25–3.87)	1.00 (Ref)	1.00 (0.79–1.27)	2.06 (1.57–2.71)	3.57 (2.68–4.76)
	p value*	< 0.001	< 0.001	-	0.994	< 0.001	< 0.001
**Model 1**	HR (95%CI)	1.37 (1.10–1.71)	2.42 (1.91–3.06)	1.00 (Ref)	0.87 (0.72–1.05)	1.32 (1.00-1.73)	2.35 (1.87–2.96)
	p value	0.006	< 0.001	-	0.155	0.049	< 0.001
**Model 2**	HR (95%CI)	1.53 (1.22–1.93)	2.17 (1.70–2.78)	1.00 (Ref)	0.88 (0.72–1.09)	1.33 (1.00-1.76)	1.99 (1.48–2.66)
	p value	< 0.001	< 0.001	-	0.235	0.051	< 0.001
**Model 3**	HR (95%CI)	1.54 (1.21–1.95)	2.14 (1.64–2.79)	1.00 (Ref)	0.90 (0.73–1.12)	1.27 (0.95–1.69)	1.98 (1.46–2.69)
	p value	0.001	< 0.001	-	0.332	0.099	< 0.001

Ref, treating the bottom group (MMA ≤ 250 nmol/L, B12 ≤ 400 pg/mL) as the reference;

*Hazard ratio per 1 unit increases of natural log-transformed MMA; # Values are weighted hazard ratio (95% confidence interval)

Model 1: adjusted for age (years, continuous), sex (female or male), and race/ethnicity (non-Hispanic white, black, Hispanic-Mexican, or other)

Model 2: additionally adjusted for smoking status (never, ever or current), physical activity (inactive, moderate, or vigorous), body mass index (< 18.5, 18.5–25, 25–30, or ≥ 30 kg/m^2^), hypertension (no/yes), diabetes (no/yes), chronic obstructive pulmonary disease (no/yes), cancer (no/yes), total cholesterol (mmol/L, continuous), High-density lipoprotein cholesterol (mmol/L, continuous), C-reactive protein (mg/dL, continuous), and estimated glomerular filtration rate (ml/min/1.73 m², continuous)

Model 3: additionally adjusted for metformin use (no/yes), ACEI/ARB use (no/yes), β-blocker use (no/yes), diuretics use (no/yes), anti-lipid use (no/yes) and anti-platelet use (no/yes)

## Discussion

In this study, we comprehensively analysed the associations between MMA and B12-related biomarkers and mortality in a prospective cohort of 1755 patients with coronary heart disease (CHD). Our finding showed that circulating MMA level was independently associated with all-cause death and cardiovascular mortality risk in patients with CHD, while serum B12 concentration, dietary B12 intake from food and B12 supplements use were not significantly associated with mortality risk. In spite of this, serum MMA was interacted with B12 for increased mortality risk. CHD patients with the combination of serum B12 > 400 pg/mL and MMA > 250 nmol/L had the highest mortality risk. Our findings firstly demonstrated that MMA accumulation but not serum or dietary B12 was associated with increased cardiovascular mortality risk among patients with CHD. That inconsistence may be related to decreased response to B12, not insufficient B12 level.

MMA, an established biomarker of B12 deficiency, represents a promising target for cardiovascular risk stratification and early intervention. Earlier investigations have proposed that MMA may function as a facilitator in neoplastic progression while also exhibiting a potential association with clinical frailty. However, no significant association was observed between MMA and cancer-related death in patients with CHD. The limited number of patricians and cancer-related deaths might explain this [22, 23]. Several cross-sectional studies have suggested that MMA is a potential risk factor for cardiovascular disease (CVD), although small sample sizes may have limited their effectiveness [24, 25]. Compared to healthy population, individuals with cardiovascular disease, including acute myocardial infarction (AMI) and acute heart failure had higher levels of circulating MMA [24, 25]. It is imperative to explore novel strategies aimed at reducing MMA levels, considering the limited efficacy of B12 supplementation revealed by our findings. Unraveling the underlying mechanisms and identifying innovative approaches to effectively manage B12 level to mitigate the concomitant risks of mortality assume paramount importance in this context.

Preclinical study has suggested that vitamin B12 has potential cardiovascular metabolic benefits, including reducing oxidative stress and improving insulin resistance [26]. However, clinical studies on high-dose B12 supplementation have produced suboptimal results [11, 27, 28]. For the first time, we attempt to comprehensively investigate the relationship between B12-related biomarkers, MMA and cardiovascular mortality risk in CHD patients. Serum MMA was significantly associated with all-cause and cardiovascular mortality, while serum B12, dietary intake and B12 supplementation showed no significant correlation with mortality risk. According to the intersection point, we further conducted the Landmark analysis to assess the mortality risk occurring before and after 9 years of follow-up across tertiles of serum B12. Despite the borderline significance observed between the groups during the 9-year period, it is important to interpret the results cautiously due to the lack of significant difference in the multivariable-adjusted Cox regression analysis. We cannot completely rule out the possible relationship between B12 level and mortality due to the limited sample size, at least this relationship appears weaker than the association of MMA with mortality. This is consistent with our previous reports that MMA has potential advantages as a potential prognostic indicator. Serum MMA predicted a 10-year risk of mortality in adults with cardiovascular disease better than CRP [9].

Although prior studies have reported a positive association between serum B12 levels and increased risk of cancer or related mortality in general population and critically ill medical patients, attributing deleterious effects solely to excess B12 may be inappropriate [6, 29, 30]. Presently, there is no strong evidence regarding the relationship between B12 intake and adverse events [29]. The positive association between serum B12 and mortality risk might stem from compensatory elevation of B12 levels in response to impaired B12 sensitivity. This point could be elucidated by the lack of response to B12 treatment in congenital methylmalonic aciduria resultant from MMUT gene mutations.

The pathological state of both elevated B12 and MMA is predominantly described in diabetes and geriatric patients, termed functional B12 deficiency. However, even with supplementation utilizing activated B12 forms such as methylcobalamin and adenosylcobalamin injections, discernible clinical benefits in terms of cardiovascular health remain unsubstantial. The term “impaired sensitivity to B12” more aptly captures this condition than “functional B12 deficiency,“ encompassing a broader portrayal of the potential underlying pathological physiological process involving decreased responsiveness to B12 treatment, including B12 absorption, transport, intracellular metabolism, and mitochondrial MMA metabolism.

Although we did not observe a correlation between serum B12 and mortality risk in CHD patients, our study for the first time found that nearly a half of CHD patients with elevated MMA had adequate B12 levels (> 400 pg/mL). Furthermore, patients with higher levels of both MMA and B12 had an elevated risk of mortality. This suggests that impaired B12 sensitivity is common in CHD, and high-dose B12 supplementation may provide limited benefits for reducing cardiovascular risk. This is consistent with previous negative results of B12 intervention within clinical trials. Our findings expanded the understanding of B12 management in patients with CHD. Beyond simply concentrating on B12 deficiency, it becomes paramount to address the matter of declined sensitivity to B12. Elucidation of the mechanisms underlying this association remains further exploration. This may help address the challenges of suboptimal benefits of B12 supplementation and explore new methods for reducing MMA levels and improving cardiovascular health.

This study has several strengths. The utilization of a nationally representative sample enhances the generalizability of the findings to a broader population. The methods used to collect variables in the research data have been validated to minimize bias and the data in the final analysis has been thoroughly adjusted. Our study has several limitations. Firstly, the sample size may limit the investigation of the correlation between serum B12, dietary intake, and risk of mortality in CHD patients and we did not investigate the form and dose of B12 supplements. However, our results suggest that impaired sensitivity to B12 supplementation is common in patients with CHD and is associated with a significantly higher risk of mortality compared to B12 levels. Secondly, this study is an observational study, and therefore, the causality could not be concluded. Further exploration is needed to understand the mechanisms underlying this association. Thirdly, we only included patients with a prior diagnosis of CHD. Subclinical CHD requires screening and additional investigation. Fourthly, despite adequate adjustment, unknown confounding factors cannot be ruled out.

## Conclusion

Although MMA was considered a marker of vitamin B12 deficiency, we found that MMA accumulation but not serum or dietary B12 was associated with increased risk of cardiovascular mortality among patients with CHD. This paradox may be related to decreased response to B12. Our findings may provide novel insights into MMA and B12 management in patients with CHD.

### Electronic supplementary material

Below is the link to the electronic supplementary material.

Supplementary Material 1

## Data Availability

The datasets of NHANES are available on reasonable request from the website. (https://www.cdc.gov/nchs/nhanes/index.htm.)
