# Supplementary material for: Methylmalonic acid, vitamin B12, and mortality risk in patients with preexisting coronary heart disease: a prospective cohort study
Source: Nutr J. 2023 Nov 29;22:63. doi: 10.1186/s12937-023-00900-6 (PMC10685606; doi:10.1186/s12937-023-00900-6)
Supplement: Supplementary file 1 — Supplementary Material 1 [file 12937_2023_900_MOESM1_ESM.docx]

**Supplementary data**

## Supplementary Table 1. Partial correlation of methylmalonic acid with cardiometabolic biomarkers in NHANES 1999-2004

| **Variables** | **R** | **p value** |
| --- | --- | --- |
| BMI, kg/m^2^ | 0.006 | 0.814 |
| Waist circumference, cm | 0.010 | 0.701 |
| Creatinine, µmol/L | 0.388 | <0.001 |
| Blood urea nitrogen, mmol/L | 0.293 | <0.001 |
| Glucose, mmol/L | 0.076 | 0.002 |
| HbA1c, % | 0.066 | 0.006 |
| C-peptide, nmol/L | 0.229 | <0.001 |
| Insulin, pmol/L | 0.074 | 0.035 |
| HOMA-IR index | 0.116 | 0.002 |
| Hcy, µmol/L | 0.482 | <0.001 |
| Triglycerides, mmol/L | 0.078 | 0.001 |
| Cholesterol, total, mmol/L | -0.054 | 0.026 |
| HDL-C, mmol/L | -0.102 | <0.001 |
| LDL-C, mmol/L | -0.084 | 0.021 |
| Systolic BP, mmHg | -0.015 | 0.537 |
| Diastolic BP, mmHg | -0.087 | <0.001 |
| eGFR, mL/min per1.73m² | -0.405 | <0.001 |
| CRP, mg/dL | -0.001 | 0.978 |
| B12, pg/mL | -0.291 | <0.001 |
| Serum folate, nmol/L | -0.028 | 0.358 |

Partial correlation coefficients were estimated by the Pearson analysis corrected for age, sex, and race. BMI, body mass index; BP, blood pressure; ALT, alanine aminotransferase; AST, Aspartate transaminase; eGFR, estimated glomerular filtration rate; CRP, C-reactive protein; HDL-C, high-density lipoprotein cholesterol; HOMA-IR, Homeostasis Model Assessment-Insulin Resistance; LDL-C, low-density lipoprotein cholesterol; B12, Vitamin B12.

## Supplementary Table 2. The association of methylmalonic acid with stroke and cancer.

|  |  |  | **MMA, nmol/L** | | |  |
| --- | --- | --- | --- | --- | --- | --- |
| **Cause of death** | **log MMA* (n=1755)** | **p value** | **T1 (n=592)** | **T2 (n=579)** | **T3 (n=584)** | **p trend** |
| Stroke |  |  |  |  |  |  |
| Deaths/ person-yrs | 56/15877 | - | 10/6782 | 24/5060 | 22/4034 | - |
| Crude | 1.76 (1.18-2.62) | 0.006 | 1 (ref.) | 2.77 (1.14-6.75) | 3.32 (1.44-7.68) | 0.002 |
| Model 1 | 1.08 (0.60-1.94) | 0.598 | 1 (ref.) | 1.55 (0.60-3.97) | 1.67 (0.67-4.18) | 0.267 |
| Model 2 | 1.38 (0.56-3.45) | 0.645 | 1 (ref.) | 1.80 (0.58-5.60) | 2.41 (0.75-7.78) | 0.121 |
| Model 3 | 1.46 (0.56-3.85) | 0.714 | 1 (ref.) | 1.87 (0.60-5.82) | 2.58 (0.77-8.61) | 0.103 |
| Cancer |  |  |  |  |  |  |
| Deaths/ person-yrs | 161/15877 | - | 59/6782 | 54/5060 | 48/4034 | - |
| Crude | 1.40 (0.95-2.06) | 0.087 | 1 (ref.) | 1.01 (0.64-1.59) | 1.34 (0.81-2.21) | 0.289 |
| Model 1 | 1.09 (0.68-1.75) | 0.710 | 1 (ref.) | 0.77 (0.49-1.22) | 0.97 (0.56-1.70) | 0.858 |
| Model 2 | 1.18 (0.68-2.09) | 0.545 | 1 (ref.) | 0.83 (0.51-1.36) | 1.06 (0.54-2.11) | 0.919 |
| Model 3 | 1.10 (0.62-1.99) | 0.733 | 1 (ref.) | 0.80 (0.49-1.31) | 0.96 (0.48-1.92) | 0.856 |

*Hazard ratio per 1 unit increases of natural log-transformed MMA; # Values are weighted hazard ratio (95% confidence interval).

Model 1: adjusted for age (years, continuous), sex (female or male), and race/ethnicity (non-Hispanic white, black, Hispanic-Mexican, or other).

Model 2: additionally adjusted for smoking status (never, ever or current), physical activity (inactive, moderate, or vigorous), body mass index (<18.5, 18.5–25, 25–30, or ≥30 kg/m^2^), hypertension (no/yes), diabetes (no/yes), chronic obstructive pulmonary disease (no/yes), cancer (no/yes), total cholesterol (mmol/L, continuous), High-density lipoprotein cholesterol (mmol/L, continuous), C-reactive protein (mg/dL, continuous), Vitamin B12 (B12, continuous) and estimated glomerular filtration rate (ml/min/1.73m², continuous).

Model 3: additionally adjusted for metformin use (no/yes), ACEI/ARB use (no/yes), β-blocker use (no/yes), diuretics use (no/yes), anti-lipid use (no/yes) and anti-platelet use (no/yes).

## Supplementary Table 3. The associations between serum and dietary B12 and mortality risk in adults with CHD

|  | **Serum B12, pg/ml** | | | |
| --- | --- | --- | --- | --- |
| **All-cause mortality** | **T1 (n=581)** | **T2 (n=582)** | **T3 (n=580)** | **p trend** |
| Deaths/ person-yrs | 340/4188.2 | 309/4136.5 | 324/3588.5 |  |
| Crude | 1.00 (Ref) | 0.84 (0.67-1.06) | 1.06 (0.85-1.31) | 0.667 |
| Model 1 | 1.00 (Ref) | 0.86 (0.72-1.04) | 0.99 (0.82-1.21) | 0.913 |
| Model 2 | 1.00 (Ref) | 0.87 (0.72-1.06) | 0.96 (0.78-1.18) | 0.635 |
| Model 3 | 1.00 (Ref) | 0.90 (0.74-1.11) | 0.98 (0.80-1.20) | 0.835 |
|  | **Dietary B12 intakes from foods, mg/day** | | | |
| **All-cause mortality** | **T1 (n=559)** | **T2 (n=560)** | **T3 (n=556)** | **p trend** |
| Deaths/ person-yrs | 299/3856.2 | 332/3780.9 | 299/4021.3 |  |
| Crude | 1.00 (Ref) | 1.10 (0.89-1.35) | 0.83 (0.68-1.01) | 0.055 |
| Model 1 | 1.00 (Ref) | 1.07 (0.88-1.30) | 0.94 (0.78-1.15) | 0.554 |
| Model 2 | 1.00 (Ref) | 1.01 (0.81-1.25) | 0.88 (0.71-1.10) | 0.243 |
| Model 3 | 1.00 (Ref) | 1.01 (0.81-1.25) | 0.91 (0.73-1.14) | 0.388 |
|  | **B12 supplement use** | | |  |
| **All-cause mortality** | **No use (n=1114)** | **Use (n=643)** | **p value** |  |
| Deaths/ person-yrs | 608/7607.3 | 372/4436.5 |  |  |
| Crude | 1.00 (Ref) | 1.07 (0.90-1.28) | 0.449 |  |
| Model 1 | 1.00 (Ref) | 0.90 (0.77-1.04) | 0.146 |  |
| Model 2 | 1.00 (Ref) | 0.94 (0.80-1.11) | 0.467 |  |
| Model 3 | 1.00 (Ref) | 0.97 (0.81-1.15) | 0.699 |  |

Ref, treating the bottom group as the reference.

*Hazard ratio per 1 unit increases of natural log-transformed MMA; # Values are weighted hazard ratio (95% confidence interval).

Model 1: adjusted for age (years, continuous), sex (female or male), and race/ethnicity (non-Hispanic white, black, Hispanic-Mexican, or other).

Model 2: additionally adjusted for smoking status (never, ever or current), physical activity (inactive, moderate, or vigorous), body mass index (<18.5, 18.5–25, 25–30, or ≥30 kg/m^2^), hypertension (no/yes), diabetes (no/yes), chronic obstructive pulmonary disease (no/yes), cancer (no/yes), total cholesterol (mmol/L, continuous), High-density lipoprotein cholesterol (mmol/L, continuous), C-reactive protein (mg/dL, continuous), Vitamin B12 (B12, continuous) and estimated glomerular filtration rate (ml/min/1.73m², continuous).

Model 3: additionally adjusted for metformin use (no/yes), ACEI/ARB use (no/yes), β-blocker use (no/yes), diuretics use (no/yes), anti-lipid use (no/yes) and anti-platelet use (no/yes). Models for B12 supplements use were adjusted for dietary B12 intake from foods (continuous) and vice versa.

## Supplementary Table 4. Stratification analysis for the HRs of all-cause mortality in NHANES.

| **Subgroup** | **HR** | **p value** | **p for interaction** |
| --- | --- | --- | --- |
| **Age, years** |  |  | **0.527** |
| <65 | 1.74 (1.20-2.52) * | 0.004 |  |
| ≥65 | 1.84 (1.53-2.23) | <0.001 |  |
| **Sex** |  |  | **0.817** |
| Female | 1.99 (1.51-2.63) | <0.001 |  |
| Male | 1.65(1.32-2.07) | <0.001 |  |
| **Current smoking** |  |  | **0.060** |
| No | 1.81 (1.46-2.25) | <0.001 |  |
| Yes | 1.79 (1.38-2.32) | <0.001 |  |
| **BMI, kg/m²** |  |  | **0.307** |
| <30 | 1.76(1.42-2.17) | <0.001 |  |
| ≥30 | 1.70 (1.32-2.17) | <0.001 |  |
| **Diabetes** |  |  | **0.019** |
| Yes | 2.51(1.99-3.16) | <0.001 |  |
| No | 1.63(1.31-2.03) | <0.001 |  |
| **eGFR, ml/min/1.73m^2^** |  |  | **0.418** |
| ≥60 | 1.61 (1.30-2.01) | <0.001 |  |
| <60 | 2.25(1.74-2.90) | <0.001 |  |

Values are hazard ratios (95% confidence interval). HRs (95%CI) were assessed using weighted Cox proportional regression after adjustment for age (years, continuous), sex (female or male), and race/ethnicity (non-Hispanic white, black, Hispanic-Mexican, or other), smoking status (never, ever or current), body mass index (<18.5, 18.5–25, 25–30, or ≥30 kg/m^2^), hypertension (no/yes), diabetes (no/yes), chronic obstructive pulmonary disease (no/yes), cancer (no/yes), physical activity (inactive, moderate, or vigorous), total cholesterol (mmol/L, continuous), high-density lipoprotein cholesterol (mmol/L, continuous), C-reactive protein (mg/dL, continuous), and estimated glomerular filtration rate (ml/min/1.73m², continuous), metformin use (no/yes), ACEI/ARB use (no/yes), β-blocker use (no/yes), diuretics use (no/yes), anti-lipid use (no/yes) and anti-platelet use (no/yes) except for stratification factor.

## Supplementary Figure 1 Flow chart


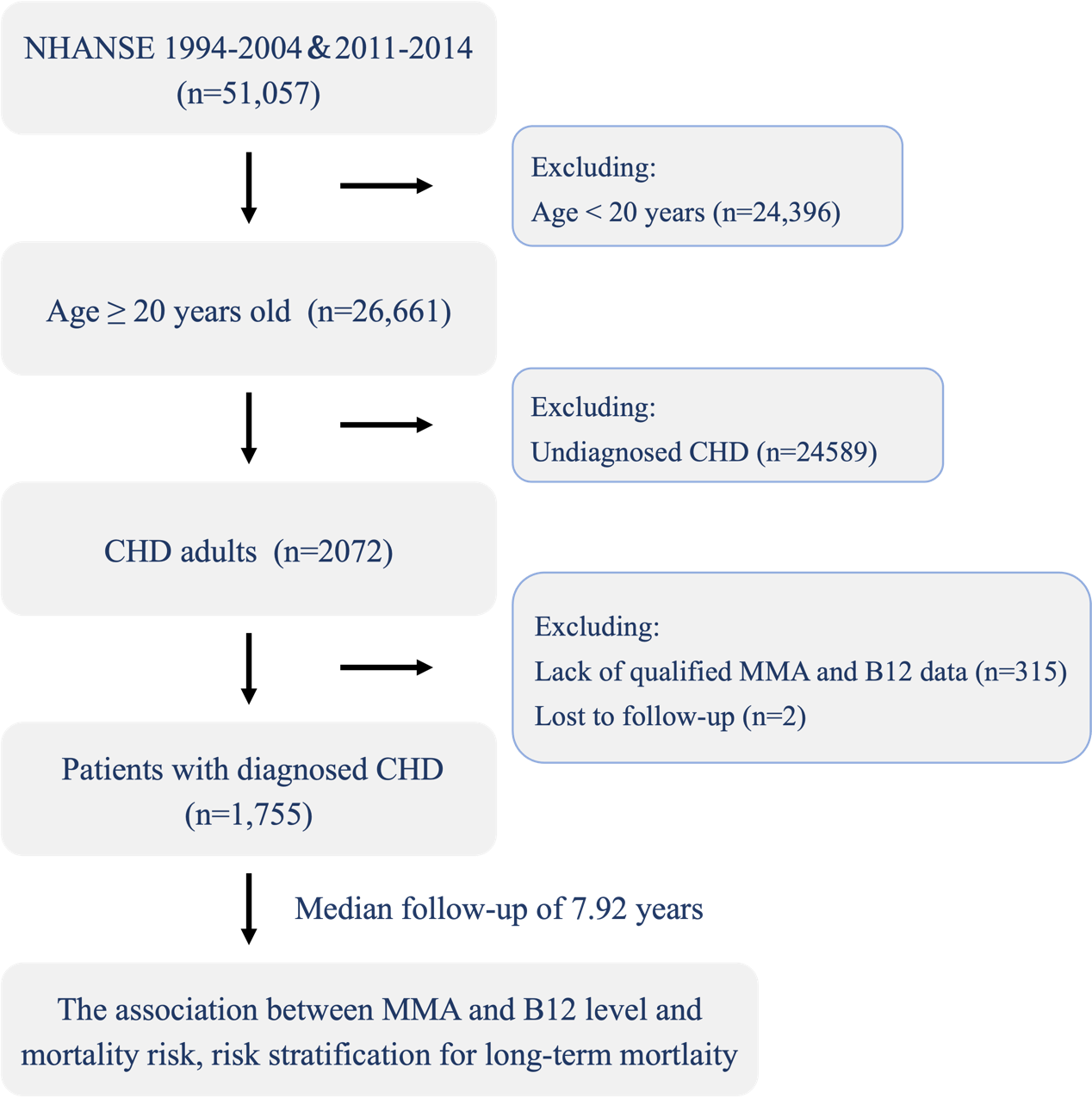


CHD, coronary heart disease

## Supplementary Figure 2. Accumulative cause-specific mortality by baseline methylmalonic acid strata


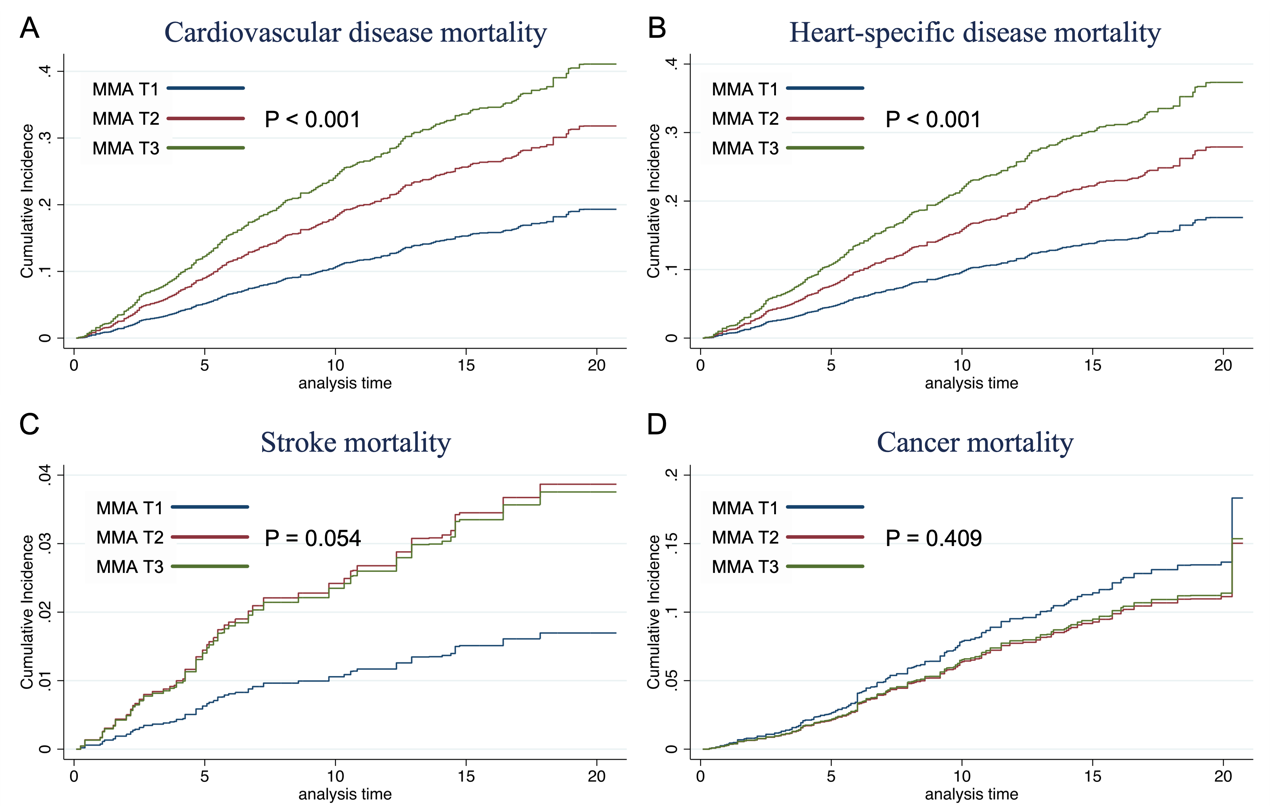


A, Cardiovascular disease mortality of the three groups during 16 years; B, Heart-specific disease mortality in NHANES of the three groups during 16 years; C, Stroke mortality of the three groups during 16 years; D, Cancer mortality of the three groups during 16 years. The accumulative incidence of cause-specific mortality was calculated by cumulative incidence function with competing risk regression model. Y-axis shows the absolute risk of cumulative mortality rate (%). The X-axis shows the follow-up period (years).

## Supplementary Figure 3. The receiver-operating characteristic curve (ROC) shows MMA and CRP predicting 10-year mortality risk in CHD patients


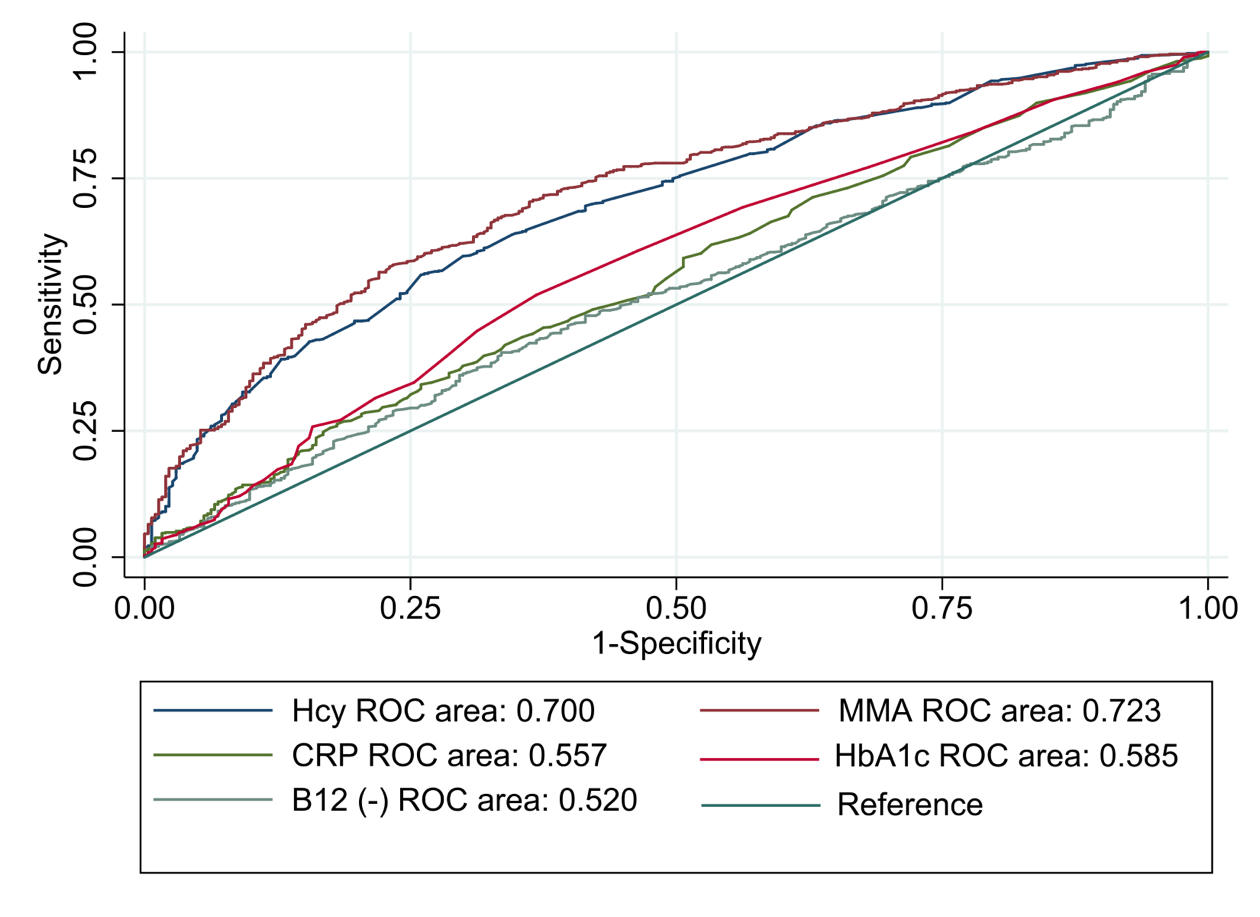


ROC area: the nonparametric estimate of the area under the empirical ROC curve is the summation of the areas of the trapezoids formed by connecting the points on the ROC curve.

**Supplementary Figure 4.** **Landmark analysis** **discriminating between a****ll-cause mortality occurring before and after 9 years of follow-up** **across level of serum vitamin B12

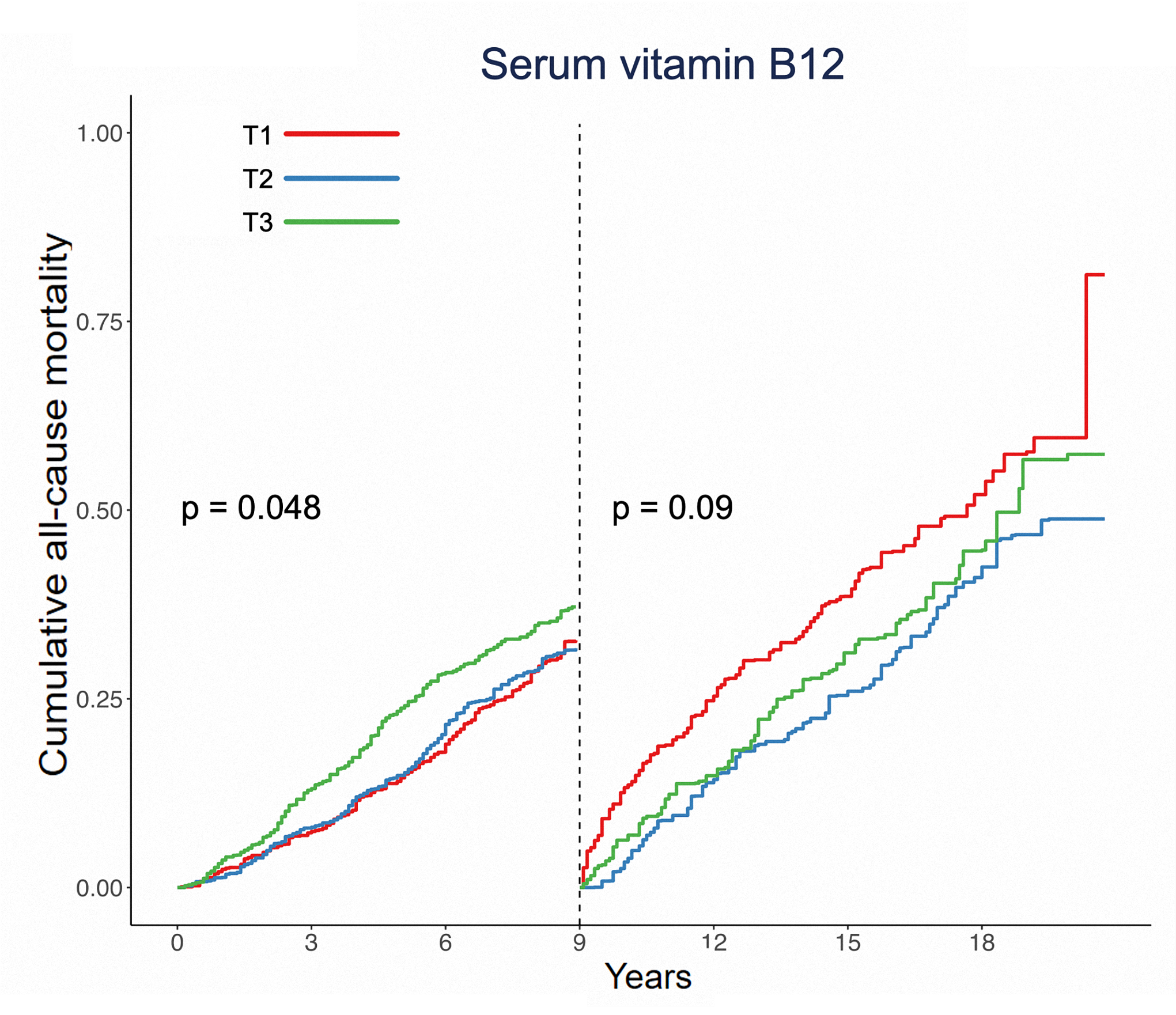
**Y-axis shows the absolute risk of cumulative mortality rate (%). P value was estimated by log-rank test. The X-axis shows the follow-up period (years). Although there is a borderline significance between groups during the 9-year follow-up period (p=0.048), the interpretations should be cautious due to the nonsignificant difference observed in the multivariable-adjusted Cox regression analysis. During the follow-up of first 9 years, the multivariable-adjusted HRs (95% CIs) across the B12 tertiles were 1.00 (reference), 1.08 (0.81–1.45), and 1.16 (0.90–1.49) for all-cause mortality (each p ≥0.239).
